# Dysregulation of autophagy and mitochondrial function in Parkinson’s disease

**DOI:** 10.1186/s40035-016-0065-1

**Published:** 2016-10-31

**Authors:** Bao Wang, Neeta Abraham, Guodong Gao, Qian Yang

**Affiliations:** 1 Department of Neurosurgery, Tangdu Hospital, The Fourth Military Medical University, No. 569 Xinsi Road, Baqiao District, Xi’an, 710038 Shaanxi Province China; 2 Department of Neurology, Beth Isreal Deaconess Medical Center, Harvard Medical School, 330 Brookline Ave, Boston, 02215 MA USA

**Keywords:** Parkinson’s disease, Autophagy, Macroautophagy, Mitophagy, Chaperone-mediated autophagy, Mitochondria,α-synuclein, PINK/Parkin, LRRK2, DJ-1

## Abstract

Parkinson’s disease (PD) is the second most common neurodegenerative disease. Increasing evidence supports that dysregulation of autophagy and mitochondrial function are closely related with PD pathogenesis. In this review, we briefly summarized autophagy pathway, which consists of macroautophagy, microautophagy and chaperone-mediated autophagy (CMA). Then, we discussed the involvement of mitochondrial dysfunction in PD pathogenesis. We specifically reviewed the recent developments in the relationship among several PD related genes, autophagy and mitochondrial dysfunction, followed by the therapeutic implications of these pathways. In conclusion, we propose that autophagy activity and mitochondrial homeostasis are of high importance in the pathogenesis of PD. Better understanding of these pathways can shed light on the novel therapeutic methods for PD prevention and amelioration.

## Background

Parkinson’s disease (PD) is the second most common neurodegenerative disease. Although most of PD cases are sporadic, more than 5 % are inherited [1]. The PD related genes mainly include (1) *SNCA*, the gene encoding α-synuclein, which is the main component of Lewy bodies [2]; (2) *LRRK2*, which encodes a multi-domain large protein. Variable LRRK2 mutants contribute to over 10 % of familial and about 3 % of sporadic PD cases [3]; (3) *PINK1* and *Parkin*, which are genes involved in mitochondrial turnover and maintenance. Their mutations are associated with early-onset PD [4]. (4) *DJ-1*, whose mutants contribute to 1–2 % of autosomal recessive PD [5].

Given the role of autophagy in the elimination of cellular dysfunctional proteins/organelles, the contribution of impaired autphagy to PD has attracted increasing interest. Accumulating evidence indicates autophagy defects are involved in the PD pathogenesis. PD related VPS35 D620N mutant inhibits autophagy and impairs the trafficking of the autophagy protein ATG9A [6]. In addition, in the substantia nigra (SN) of PD patients, aberrant autophagy activity is identified [7]. Inducing autophagy by manipulating Polo-like kinase 2 or activiting chaperone-mediated autophagy (CMA) can reduce α-synuclein aggregation [8, 9]. Finally, PD related neurotoxin 1-methyl-4-phenyl-1,2,3,6-tetrahydropyridine (MPTP) can induce autophagy through CDK5-mediated phosphorylation of endophilin B1 [10]. Rotenone could impair mitophagy to damage neurons which can be rescued by DJ-1 overexpression [11].

Recently, increasing reports support the role of mitochondrial dysfunction in the degeneration of DA neurons during PD initiation and progression. Mitochondria are double membrane organelles and participate in multiple cellular processes, including calcium homeostasis, energy supply, metabolic synthesis and apoptosis [12]. Mutations in mtDNA are more frequent in PD patients when compared with the age-matched population [13]. Interestingly, one recent study reports that α-synuclein overexpression augments mitochondrial Ca^2+^ transient by enhancing endoplasmic reticulum-mitochondria interactions and physiological levels of α-synuclein are essential to maintain mitochondrial function and morphology [14]. MPTP and rotenone can disturb mitochondrial function, impair respiratory chain complex and induce oxidative stress [15, 16]. Thus, excessive mitochondrial stress in response to environmental toxins or impaired capability of neurons to remove damaged mitochondria through mitophagy may result in the death of neurons.

This review discussed the recent findings in the relationship among several PD related genes, autophagy and mitochondrial dysfunction (Table 1).

**Table 1:** PD-related genes are involved in dysregulation of autophagy and mitochondria

α-synuclein	Macroautophagy
	Mitophagy
	CMA
PINK1/Parkin	Mitophagy
LRRK2	Macroautophagy
	CMA
	Mitochondria
DJ-1	Macroautophagy
	CMA
	Mitochondria

## Autophagy

Autophagy, which derived from the Greek words for “self-digesting”, refers to the cellular catabolic process to remove the cytosolic components, including mainly dysfunctional organelles, misfolded proteins, and surplus or unnecessary cytoplasmic contents to lysosome for digestion [17].

Autophagy can be divided into three major types: macroautophagy, microautophagy and chaperone-mediated autophagy (CMA). This classification is dictated by the way in which the cellular components are being delivered to lysosomes [17].

Macroautophagy (MA) is the most common and well conserved form of autophagy, and enables the bulk degradation of cellular contents [18]. It mainly involves five steps: initiation, nucleation, elongation, fusion, and degradation [19]. In this process, the pre-autophagosomal structure (PAS) elongates from membrane of Golgi, mitochondria or endoplasmic reticulum and sequesters the cullular contents or organelles to form the autophagosome. Subsequently, the autophagosome fuses with lysosomes to allow the cargo to be degraded [20]. Mitophagy describes the clearance of damaged mitochondria by macroautophagy. Increasing evidence prove that dysregulation of mitophagy play important roles in neurodegenerative diseases [21].

Microautophagy is the process in which cytoplastic contents are directly engulfed into lysosomes for degradation without an intermediate autophagosome [22]. Also, microautophagy is constitutionally active under normal conditions and maintains the turnover of cellular nutrients via selective degradation of proteins and organelles [23]. Unlike macroautophagy, nutritional deprivation or stress cannot activate microautphagy [24]. Currently, knowledge about the role and function of microautophagy, especially in neurodegenerative disease, is limited and further studies are needed.

CMA is characterized by the high selectivity in substrates targeting [25]. The substrates for CMA present a unique pentapeptide motif, which is known as KFERQ. Notably, this motif is a pattern recognition motif associated with the hydrophobicity and charge of amino acid residues, rather than 100 % compliance with specific amino acid residues [26]. Interestingly, post-translational modification could perfect some KFERQ-similar motif for CMA through changing the hydrophobicity and charge of amino acid residues [27]. Briefly, CMA can be divided into four main steps: recognition, unfolding, translocation and degradation [28]. Cytoplastic heat shock cognate protein 70 (Hsc70) binds to the substrates after recognizing the KFERQ motif and delivers it to lysosomal surface, where the substrates could be unfolded with the help of Hsc70 and other chaperons. Afer that, the unfolded substrate is translocated into lysosome via lysosome associated membrane protein type 2A (LAMP2A) for degradation under the lysosomal acid environment.

## Mitochondrial dysfunction in Parkinson’s disease

Although the mechanism underlying PD pathogenesis is still unclear, mitochondrial dysfunction has been increasingly confirmed to be a vital contributor. The main characterizations of mitochondrial dysfunction include ROS over-production, ATP depletion, mitochondrial DNA depletion, caspase release and electron transport complex (ETC.) enzyme defection [29]. Neurotoxins applied to establish PD models, including MPTP and rotenone, damage mitochondrial function by inhibiting complex I activity [30]. One recent study specifically knockouts *Ndufs4* gene in mice midbrain DA neurons and reports significant compromise in complex I activity. However, *Ndufs4* deficit mice show no obvious neurodegeneration, no loss of striatal innervation and no movement impairment, but with increased vulnerability to MPTP, suggesting that impaired complex I activity is involved in the pathogenesis of PD [31]. Another study shows that complex I inhibition can lead to abnormal oxidition of optic atrophy 1 (OPA1), which in turn results in mitochondrial structural abnormalities and dopaminergic neurodegeneration. All these pathogenic changes are abolished by OPA1 upregulation, hinting that OPA1 is a novel therapeutic target for PD [32].

Mitophagy means the way in which damaged mitochondria are eliminated by autophagy. Through mitophagy, cells regulate both the number and quality of mitochondria in response to the metabolic stress. One recent study using genome-wide RNAi screen identifies *sterol regulatory element binding transcription factor 1* (*SREBF1*), *F-box* and *WD40 domain protein 7* (*FBXW7*) as key regulators for PINK/Parkin pathway, which is reponsible for mitophagy activition [33]. Moreover, SREBF1 is considered as a risk locus for sporadic PD [34]. Thus, this study hints that mitophagy may act as a common mechanistic link between sporadic and autosomal recessive PD. Mutation in F-box domain-containing protein Fbxo7 is related with early-onset autosomal recessive PD. One study reports that Fbox7 is involved in mitochondrial maintainance via directly interaction with Parkin to induce mitophagy, which further confirms the importance of mitophagy in PD pathogenesis [35].

Our study shows that the myocyte enhancer factor 2D (MEF2D) is present in neuronal mitochondria and heat shock protein 70 (Hsp70) interacts with MEF2D and mediates its mitochondrial translocation. Mitochondrial MEF2D is responsible for the expression of the gene NADH dehydrogenase 6 (ND6), which encodes an essential component of the complex I. Blocking MEF2D function leads to impaired complex I activity, oxidative stress and neuronal death. More importantly, in the postmortem PD brain samples, both mitochondrial MEF2D and ND6 levels are decreased compared with age-matched controls [36]. Our further study shows that MPTP caused a significant decrease in the half-life and total level of MEF2D mRNA. Down-regulation of MEF2D mRNA alone can reduces the viability of SN4741 cells and sensitizes the cells to neurotoxin [37].

## PD-related genes are involved in dysregulation of autophagy and mitochondria

### α-synuclein

α-synuclein is encoded by *SNCA* and its fibrillar form is the major component of Lowy bodies [38]. Existence of hydrophobic non-amyloid β component domain endows α-synuclein with the propensity to aggregate [39]. Aggregated α-synuclein can trigger neuronal death in PD [40]. Thus, intensive researches focus on how to prevent or eliminate the aggregation of α-synuclein. Study show that ubiquitin proteasome system (UPS) is responsible for degrading monoubiquitinated α-synuclein, while macroautophagy is for removing deubiquitinated α-synuclein [41, 42]. Further study demonstrates, in normal condition, α-synuclein is mainly degraded by UPS [43]. Macroautophagy pathway can be activated in response to increased level of wild type or A53T mutant α-synuclein [44, 45]. However, some studies show that increased α-synuclein can impair autophagosome synthesis by inhibiting Rab1a [46]. Also, pre-formed α-synuclein aggregates compromise autophagosome clearance and are resistant to degradation by autophagy [47]. The difference may be derived from the variability of models or different levels of α-synuclein. Further studies are warranted to address such discrepancy.

Abnormal α-synuclein level can also disrupt mitophagy. In PD postmortem brain tissues, α-synuclein accumulation increases oxidative stress and disturbs mitochondrial function [48]. Moreover, both in vivo and in vitro, expression of α-isoforms of α-synuclein in neurons causes the fragmentation of mitochondria, which will eventually leads to the decline in respiration and neuronal death [49]. Over-expression of α-synuclein can occur in mitochondria and disrupt mitochondrial membrane potential by opening the mitochondrial permeability transition pore (mPTP), thereby initiating mitophagy [45]. α-synuclein can activate mPTP via interacting with either adenylate translocator (ANT) or voltage dependent anion channel (VDAC) [50, 51]. Further study found that A53T mutant could impair mitochondrial function by residing in mitochondria membrane as monomers and oligomers [52].

Increasing evidence confirms α-synuclein as a target of chaperone mediated autophagy (CMA). Hsc70 can recognize soluble α-synuclein and the affinity between Hsc70 and α-synuclein fibrils is 5-fold tighter compared with soluble α-synuclein [53]. One recent study showed that Hsc70 and Ssa1p work like a tweezer to bind two domains within α-synuclein [54]. Also, in vitro, one study shows the uptake of extracellular α-synuclein by neurons and their retrograde axonal transportation to neuronal soma. However, Hsc70 chaperones α-synuclein in the extracellular space and alleviates α-synuclein oligomer formation [55]. Activating CMA activity via up-regulating LAMP2A decreases α-synuclein turnover and protects against α-synuclein over-expression induced neurotoxicity [9]. Our study showed MEF2D, a transcription activator identified as neuronal survival factor, is the substrate of CMA. Wild type or mutant α-synuclein accumulation compromises normal turnover of MEF2D by CMA and leads to decrease in the MEF2D DNA binding ability and neuronal stress, which underlies the neuronal loss of PD [56]. Thus, enhancing CMA pathway could be a promising therapeutic strategy for PD treatment.

### PINK1/Parkin

PINK1 is a serine/threonine kinase protein and mutations in PINK1 cause a rare form of autosomal recessive PD [57]. Parkin containing ubiquitin E3 ligase can ubiquitinate multiple substrates for degradation. Mutations of Parkin lead to accumulation of its substrates and are related with early onset juvenile autosomal recessive PD [58].

Increasing evidence suggests the PINK1/Parkin pathway is essential for mitochondrial quality control. One recent study shows that Parkin ubiquitinates dynamin-related protein 1 (Drp1) to promote its degradation. Disruption of this interaction by Parkin mutation leads to the accumulation of Drp1 and mitochondrial fragmentation [59]. In *Drosophila*, both Parkin and PINK null mutants show a significant overall slowing of motichondrial protein turnover and mitophagy. Failure to remove the damaged mitochondrial proteins plays an important role in PD pathogenesis [60]. PINK1 mutation affects mitochondrial complex I activity and the maintainance of the electron transport chain, which disturbs the mitochondrial membrane potential [61]. Upregulation of Parkin protects cells against multiple stresses, including endoplasmic reticulum stress, mitochondrial stress, proteotoxicity and excitotoxicity [62, 63]. By contrast, loss of Parkin results in mitochondrial fragmentation, decreased cellular Ca^2+^ handling capability and increased cellular vulnerability to stress [64]. All the above findings demonstrate that deficiency in PINK1/Parkin pathway leads to mitochondrial dysregulation.

Recent researches on PINK1/Parkin pathway have revealed molecular details for mitochondria protection. Once the mitochondria are impaired and the membrane potential gets depolarized, PINK1 will accumulate on the outer membrane of mitochondria (OMM). A study showed dimeric PINK1 on OMM can recruit Parkin and thereby phosphorylates Parkin at Ser65 [65]. Research using mass spectrometry identified VDACs as the docking site for Parkin recruitment to the OMM [66]. After Parkin translocation to mitochondria, many OMM proteins are ubiquitinated by Parkin and in turn recruited other proteins to initiate mitophagy. Then, these Parkin labeled mitochondria are brought to lysosomes for degradation. This PINK1/Parkin signaling pathway can be positively modulated by AF-6, which is lacked in caudate/putamen and SN of sporadic PD patients [67]. Moreover, up-regulation of translocation of the OMM (TOMM) can rescue mitophagy impaired by Parkin mutations, hinting that TOMM acts as an important regulator in PINK1/Parkin mediated mitophagy [68]. Despite extensive research, how autophagy related proteins are recruited during mitophagy process is still unclear and to what extent mitophagy dysregulation contributes to PD pathogenesis remains to be investigated.

### LRRK2

Leucine-rich repeat kinase 2 (LRRK2) is one of the generic contributors to PD. As estimated, variable LRRK2 mutants contribute to over 10 % of familial and about 3 % of sporadic PD cases [69]. So far, over 50 LRRK2 mutations have been identified in PD patients. Among these, the G2019S mutation is the most common cause of autosomal dominant familial PD cases [70]. Also, G2019S mutation can be found in about 2 % sporadic PD. Thus, exploring LRRK2 pathogenicity is essential to understand the molecular mechanisms of PD.

Many reports show a relationship between LRRK2 and macroautophagy. In kidney of mouse, loss of LRRK2 leads to age-dependent bi-phasic alteration of macroautophagy activity [71]. In human neuroglioma cells, inhibition of LRRK2 kinase activity can stimulate macroautophagy in the absence of any alteration in mTOR pathway, suggesting that LRRK2 regulates autophagic activity independent of mTOR signalings [72]. Consistently, LRRK2 activates a persistent increase in autophagosome formation through a calcium associated pathway. Simultaneously, LRKR2 upregulation increases p62 and decreases the number of acidic lysosomes [73]. Moreover, G2019S mutation increases autophagic vacuoles and shortens neurite length [74]. The effects of G2019S on neurite length can be abolished by down-regulation of LC3 or Atg7 and enhanced by autophagy inducer rapamycin, hinting that autophagy plays an important role in regulation of neurite length [75]. In fibroblasts, G2019S LRRK2 mutant exacerbates MPTP-induced cell death dependent of autophagic activity [76]. Furthermore, a study showed that LRRK2 increases the number of autophagosomes by activating CaMKK/AMPK pathway [73]. In addition, G2019S mutation augments autophagic flux by MEK/ERK signaling [77].

LRRK2 and the PD-associated mutations can be degraded by CMA. In normal condition, both UPS and CMA are responsible for the degradation of wild-type LRRK2. However, G2019S LRRK2 disrupts degradation by these pathways [78, 79]. In addition, both wild-type LRRK2 up-regulation and its mutations inhibit formation of the CMA translocation complex, thereby suppressing CMA activity [80, 81]. Subsequently, the normal turnover of CMA substrates is disrupted. Although LRRK2 mutants damage CMA process, the detailed relationship with neuronal loss, especially in animal models, still needs to be further explored.

In physiological conditions, about 10 % of LRRK2 can present in the OMM, giving rise to the hypothesis that LRRK2 mutations might have influence over mitochondrial function. Study showed LRRK2 G2019S could lead to mitochondrial fragments by enhancing mitochondrial fission via phosphorylating decaprenyl diphosphate synthase subunit 2 Dlp1(Dlp1) [82]. Also, LRRK2 mutants activate dendritic mitochondrial clearance by autophagy in neurons [83].

### DJ-1

DJ-1 belongs to the peptidase C56 family and acts as a redox-responsive cytoprotective protein. PD-related DJ-1 mutants are rare and contribute to 1–2 % of autosomal recessive PD [84].

DJ-1 can serve as a regulator of autophagy. The effect of DJ-1 up-regulation on macroautophagy depends on cell type. In dopaminergic neurons, DJ-1 overexpression induces ERK-dependent mitophagy and protects against neurotoxin induced apoptosis [11]. Also, in mouse embryonic fibroblasts, loss of DJ-1 suppresses basal autophagy and disrupts mitochondrial dynamics [85]. However, in some cancer cells, DJ-1 deficiency activates autophagy via JNK signaling [86]. Further studies are needed to address the relationship between DJ-1/autophagy and DA neuronal loss in the context of PD.

As an anti-oxidative protein, DJ-1 also plays an important role in regulating mitochondrial function. In human neuroblastoma M17 cells, DJ-1 wild-type overexpression induces elongated mitochondria while DJ-1 mutants overexpression causes mitochondrial fragmentation. Interestingly, DLP1 knockdown in these mutant DJ-1 cells rescues mitochondrial morphology and function [87]. Also, in DJ-1 knockout cells, autophagy degradation was impaired and defective mitochondria accumulated [88]. Loss of DJ-1 leads to mitochondrial phenotypes including reduced membrane potential, increased fragmentation and accumulation of autophagic markers. Supplementing DJ-1-deficient cells with glutathione reverses both mitochondrial and autophagic changes suggesting that DJ-1 may act to maintain mitochondrial function during oxidative stress [89].

In our recent study [90], we found that DJ-1, harboring CMA specific motif, is a direct substrate of CMA and mutation inside the motif can disturb the degradation of DJ-1 through CMA. In addition, we showed UPS is not the primary mechanism responsible for DJ-1 degradation. More interestingly, CMA preferentially clears the oxidatively injured DJ-1 and the extensive accumulation of the oxidized DJ-1 monomer following a reduction of lysosomal and CMA activity affects the formation or alters the balance of DJ-1 dimer. Furthermore, CMA-DJ-1 pathway plays a critical role in maintaining mitochondrial morphology and function under stress and protects against PD related neurotoxins induced cytoxicity. Our findings suggest that CMA/DJ-1 pathway is vital for mitochondrial homeostasis and dysregulation of this pathway may explain the neuronal loss during PD pathogenesis.

## Recent findings on autophagy from PD human tissues

Compared with age-matched AD and control brain samples, CMA markers LAMP2A and Hsc70 are significantly decreased in SN and amygdala of PD brains, hinting the role of autophagy in PD pathogenesis [91]. Furthermore, in peripheral blood mononuclear cells from PD patients, Hsc70 protein level is decreased in all PD groups, while glucocerebrosidase protein level is reduced only in the genetic PD groups [92]. This study suggests that Hsc70 and glucocerebrosidase may serve as a screening tool for PD diagnosis. In addition, lysosomal glucocerebrosidase protein level and enzyme activity are selectively reduced in the region with increased α-synuclein inside the PD brain of early stage. The loss of lysosomal glucocerebrosidase is directly related to reduced lysosomal CMA activity, increased α-synuclein and decreased ceramide, which suggests that compromise in lysosomal function contributes to PD pathology [93]. One study also shows that the fibroblasts from PD patients with the mutation G2019S have higher level of autophagy compared with wild type fibroblasts, which contributes to the more vulnerability to MPP^+^ [76]. However, another study using the primary cultured fibroblasts from sporadic PD patients detects no changes in autophagy [94]. Further, one study uses iPSC-derived DA neurons from PD patients with GBA1 mutation and finds that these neurons has autophagic and lysosomal defects as well as increase in glucosylceramide and α-synuclein levels [95]. Together, these findings provide evidence for a link between autophagic/lysosomal system and PD pathogenesis.

## Therapeutic implications

Currently, there is no effective treatment to halt the progression of neuronal loss in PD. Thus, developing effective therapeutic strategy will be of great importance and will likely result from a better understanding the relationship between macroautophagy, CMA, mitochondrial homeostasis and PD pathogenesis.

First, since PD is characterized by the presence of abnormal protein aggregation, enhancing macroautophagy to remove the redundant proteins and/or dysfunctional organelles is being considered as a potential therapeutic strategy. In vivo, enhancing autophagic activity through TFEB or Beclin-1 overexpression protects nigral neurons against α-synuclein-induced toxicity [96]. Coexpression of beclin-1 activated autophagy, reduced accumulation of α-synulein, and ameliorated associated neuritic alterations. The above data support that beclin-1 could be a promising molecular target for PD treatment. In addition, some autophagy-inducing chemical agents have been shown to decrease α-synuclein levels in cell and animal PD models [97, 98].

Furthermore, modulating CMA activity could provide benefits for PD treatment. In SH-SY5Y cells, generic overexpression of LAMP2A induces CMA activity and protectes DA neurons against α-synuclein neurotoxicity [9]. Moreover, in rat SN, overexpression of LAMP2A via lentivirus can reduce total levels of α-synuclein and ameliorate α-synuclein induced neuronal degeneration and stratal terminal loss [9]. Also, our data demonstrated restoration of CMA activity is essential to maintain the normal function of neuronal survival factor MEF2D, thereby regulating ND6 transcription and promoting mitochondrial complex I activity, which protect DA neurons from MPTP induced neurotoxicity [36, 37]. Furthermore, retinoic acid derivatives can activate CMA to protect cells against proteotoxicity and oxidative stress [99].

Finally, considering the contribution of mitochondrial dysfunction to the pathogenesis of PD, restoring it might be a way to prevent or treat PD. The first attempt is to enhance mitophagy. In drosophila, overexpression of Parkin increases mitochondrial activity, reduces proteotoxicity and extends lifespan [100]. Also, another possible way is modulate mitochondrial biogenesis, which would rejuvenate mitochondrial pool. The peroxisome proliferator-activated receptor gamma, coactivator 1 alpha (PGC-1α) is a potential target. PGC-1α positively regulates the expression of genes required for mitochondrial biogenesis and the cellular antioxidant responses. Also, expression of PGC-1α-regulated genes is low in SN neurons in early PD and overexpression of PGC-1α could suppress ROS and neurodegeneration [101]. Thus upregulation of PGC-1α is a candidate neuroprotective strategy in PD. However, targeted up-regulation of PGC-1α via adeno-associated virus (AAV) in SN region of mice showed detrimental effect [102, 103]. Thus, further studies need to clarify this discrepancy.

## Conclusion

### Future challenges

In our opinion, autophagy activity and mitochondrial homeostasis are of high importance in the pathogenesis of PD. Better understanding of the molecular interaction between autophagy and mitochondria function can give rise to novel therapeutic methods for PD prevention and amelioration. Since hypofunctional autophagy lead to protein/organelles accumulation while excessive autophagy activation can cause cell damage even autophagic cell death. The future challenges are to determine how to optimize autophagic activity to protect neurons during PD treatment. Also, challenges exist in identifying the common molecular pathway underlying the pathogenesis of multiple neurodegenerative diseases as well as the detailed molecular interaction between autophagy and mitochondrial dysfunction, especially in PD.

## Abbreviations

ANT: Adenylate translocator
CMA: Chaperone-mediated autophagy
DA: Dopaminergic
DRP1: GTPase dynamic related protein 1
ETC.: Electron transport complex
LAMP2A: Lysosome associated membrane protein type 2A
LRRK2: Leucine-rich repeat kinase 2
MA: Macroautophagy
MFN1/2: Mitofusins 1/2
Miro: Mitochondrial Rho GTPases
MPP: Mitochondrial processing protease
MPTP: 1-methyl-4-phenyl-1,2,3,6-tetrahydropyridine
mPTP: Mitochondrial permeability transition pore
mtDNA: Mitochondrial DNA
ND6: NADH dehydrogenase 6
OMM: Outer membrane of mitochondria
PARL: Presenilin-associated rhomboid-like protease
PAS: Pre-autophagosomal structure
PD: Parkinson’s disease
PGC-1α: peroxisome proliferator-activated receptor gamma, coactivator 1 alpha
PINK1: PTEN induced putative kinase 1
ROS: Reactive oxygen species
SN: Substantia nigra
UPS: Ubiquitin proteasome system
VDAC: Voltage dependent anion channel
VTA: Ventral segmental area
